# Insights into Accelerated MRI Protocols for Pediatric Brain Assessment in Emergency Cases

**DOI:** 10.3390/diagnostics16050681

**Published:** 2026-02-26

**Authors:** Josef Gabriel Kendel, Benjamin Bender, Georg Gohla, Andrea Bevot, Till-Karsten Hauser, Ulrike Ernemann, Christer Ruff

**Affiliations:** 1 Department of Diagnostic and Interventional Neuroradiology, University of Tübingen, Hoppe-Seyler-Str. 3, 72076 Tübingen, Germany; 2 Neuropediatrics, General Pediatrics, Diabetology, Endocrinology and Social Pediatrics, University Hospital Tübingen, University of Tübingen, Hoppe-Seyler-Str. 3, 72076 Tübingen, Germany

**Keywords:** magnetic resonance imaging, pediatric emergency brain MRI, deep learning, image reconstruction, parallel imaging, simultaneous multi-slice, single-shot echo-planar imaging, multi-shot echo-planar imaging

## Abstract

Two accelerated magnetic resonance imaging (MRI) protocols for pediatric brain imaging, GOBrain and Deep Resolve Swift Brain, developed by Siemens Healthineers (Erlangen, Germany), were evaluated in a series of clinically relevant pediatric cases at 3 Tesla. Pediatric patients are particularly prone to motion, may be uncooperative, and often require sedation, especially in emergency settings. Consequently, there is a persistent clinical demand for fast brain MRI protocols that provide diagnostically sufficient image quality while minimizing examination time. Contemporary turbo spin-echo (TSE)-based clinical protocols commonly integrate parallel imaging (PI) and simultaneous multi-slice (SMS) techniques to achieve substantial reductions in scan time. Recent advances in three-dimensional volumetric encoding, compressed sensing, and deep learning (DL)-based reconstruction have further mitigated geometry-factor-related noise amplification, enabling higher acceleration factors (GOBrain). In parallel, echo-planar imaging (EPI) has emerged as a promising approach for ultrafast multi-contrast imaging. To overcome the limitations of single-shot EPI, a multi-shot EPI-based brain MRI protocol combined with the DL-based reconstruction method Deep Resolve Swift Brain has been developed. This approach leverages the efficiency of EPI while improving image quality. Using these accelerated protocols, a comprehensive diagnostic multi-contrast brain MRI examination, particularly suited to triage and emergency imaging, can be completed in minutes. This case overview, including therapy-related leukencephalopathy in acute lymphoblastic leukemia (ALL), a brain abscess, traumatic diffuse axonal injury (DAI), a posterior circulation infarction due to vertebral artery dissection, leuokostasis syndrome, and a posterior fossa tumor with obstructive hydrocephalus, demonstrates the potential clinical feasibility of both protocols in pediatric neuroimaging. Both protocols position them as supplementary options alongside established imaging protocols, while dedicated high-resolution protocols might remain necessary for subtle pathological findings, such as focal cortical dysplasia, and for neuronavigation until larger comparative studies are available.

**Figure 1:**
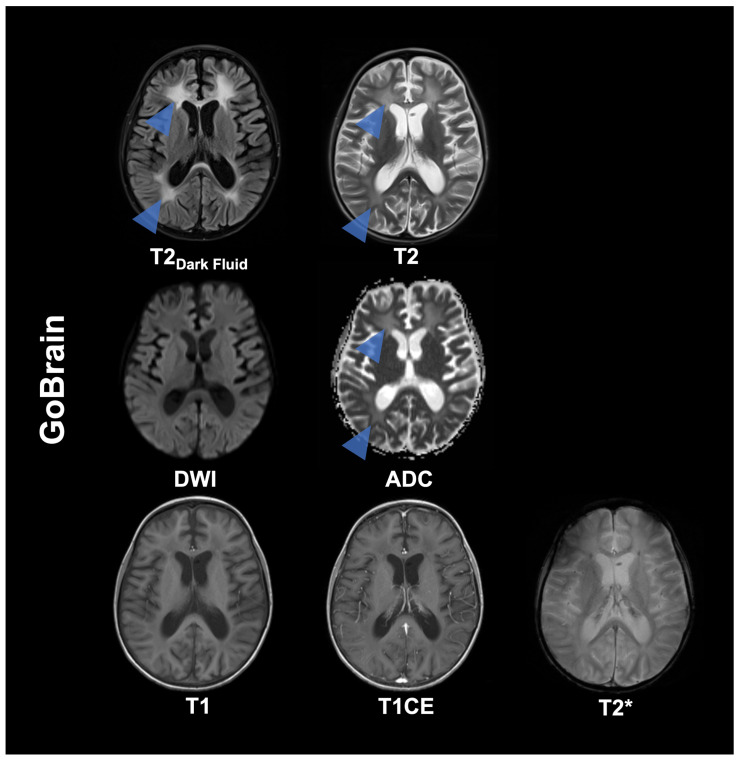
Magnetic resonance imaging (MRI) is a safe, effective technique for pediatric brain imaging and is widely used to diagnose and monitor neurologic conditions in children. It provides high-quality images with excellent soft-tissue contrast and avoids exposure to ionizing radiation. Nevertheless, MRI procedures generally require a greater time commitment than other imaging modalities, such as computed tomography (CT), which may necessitate sedation or general anesthesia. These procedures carry inherent risks and potential complications [1]. This has led to initiatives to reduce MRI acquisition times. MRI techniques encompass parallel imaging (PI) and simultaneous multi-slice (SMS) [2,3,4]. Advances in compressed sensing, 3D volumetric encoding schemes, and deep-learning reconstruction (DLR) techniques have mitigated the geometry-factor (g-factor) noise amplification in standard PI and SMS techniques, thereby providing additional acceleration and further reducing examination times [5,6,7,8,9,10]. The development of abbreviated MRI protocols has the potential to offer practical solutions by reducing the time required for MRI scans. The use of fast-brain MRI in children with shunt-dependent hydrocephalus, who require frequent serial imaging throughout their lifetimes, represents a pioneering advancement, sparing patients from unnecessary ionising radiation, which over time might theoretically lead to increased tumor risk due to their cumulative effect [2,11,12,13,14]. Recent advancements in MRI technology have further reduced acquisition times for ultrafast MRI protocols, enabling essential sequences (T1-weighted imaging (T1), T2-weighted imaging (T2), diffusion-weighted imaging (DWI), cerebrospinal fluid suppressed T2-weighted imaging (fluid attenuated inversion recovery (FLAIR) or T2 Dark Fluid), and T2*-weighted imaging (T2*)) to be acquired within 5 min, with some studies and protocols achieving times as short as 1–2 min. These developments in MRI sequences aim to expand MRI use for multiple indications, including macrocephaly, intracranial cysts, tumors, infections, screening for certain structural congenital and non-congenital anomalies, and postoperative follow-up. However, the extant research is limited, and studies focusing on a specific clinical picture are published with a delay. These studies evaluate the utility of fast and ultrafast MRI for detecting pathologies frequently encountered in routine clinical practice, especially in pediatric populations [15,16,17]. The objective of this study is to present pediatric cases and imaging examples of two fast and ultrafast MRI protocols, namely GOBrain (5:55 min) and Deep Resolve Swift Brain (1:59 min), to demonstrate the utility and feasibility of these protocols in clinical practice. The two MRI protocols under consideration were developed by Siemens Healthineers (Erlangen, Germany) and employ different technical strategies, as described above, to accelerate pediatric brain MRI and mirror technical developments over time. The development of faster MRI protocols represents a general trend pursued by other manufacturers, with no major studies published to date. This case overview is intended to provide an insight into modern accelerated imaging for children and is not intended to be a systematic comparative study with quantitative and qualitative evaluation. The objective is, therefore, not to provide a direct comparison, a benchmarking analysis, or to assert the superiority of one vendor’s approach over another. Imaging of GOBrain was performed on a 3T MAGNETOM Prisma Fit (Siemens Healthineers, Erlangen, Germany) and Deep Resolve Swift Brain on a 3T Magnetom Vida Fit (Siemens Healthineers, Erlangen, Germany). Detailed image acquisition parameters of both protocols are listed in Supplementary Table S1 and a case overview is presented in Supplementary Table S2. The GOBrain protocol consists of axial T2-weighted Dark Fluid, T2-weighted, diffusion-weighted, T1-weighted, and T2*-weighted sequences. The following key technical advances have enabled GOBrain: parallel imaging with high-channel count coils and routine 3T MRI [2,18,19]. A central design objective was to preserve the visual characteristics of existing images, particularly contrast and resolution, to facilitate standard radiological interpretation of routine examinations. Concurrently, several sequences were optimized to mitigate EPI-related susceptibility artefacts and geometric distortions by lowering EPI factors and reducing inter-echo spacing. The GOBrain images of the presented 11-year-old male patient with a history of acute lymphoblastic leukemia (ALL) exhibited a reduced general condition. The study demonstrates therapy-related leukoencephalopathy, with symmetric hyperintensities on T2 Dark Fluid and T2-weighted images in the periventricular and deep white matter (blue arrowheads), with relative preservation of the subcortical U-fibers—an established late effect of ALL therapy. Such abnormalities have been observed to persist throughout a patient’s lifetime and have been associated with gliosis, cortical atrophy, and ventricular enlargement. GOBrain is a rapid, comprehensive brain MRI that takes approximately 5 min to complete. The CSF-suppressed T2-weighted contrast is obtained using a fast turbo spin-echo (TSE) FLAIR acquisition accelerated with GeneRalized Autocalibrating Partial Parallel Acquisition (GRAPPA) factor 2, completing in under 2 min. In conventional T2-weighted imaging, a TSE sequence with a high turbo factor is imperative. This enables the collection of multiple k-space lines per repetition time (TR), thereby facilitating expedited acquisition while maintaining diagnostic efficacy. This T2-weighted sequence is further accelerated using GRAPPA with an acceleration factor of 3, synthesizing missing k-space lines from multi-coil sensitivity information, thereby preserving image quality despite undersampling. In conjunction with negligible decreases in matrix size, this facilitates whole-brain T2-weighted imaging in approximately one minute. DWI is performed with single-shot EPI (ssEPI) at b = 800 s/mm^2^. It incorporates in-plane GRAPPA to decrease echo spacing and shorten the echo train, thereby reducing susceptibility-related geometric distortion. The axial T1-weighted acquisition employs a 2D FLASH gradient-echo sequence as a time-saving alternative to spin-echo imaging. The GOBrain sequence has been developed for detecting hemorrhage. It incorporates a T2*-sensitive ssEPI sequence that collects full k-space after a single radio frequency excitation and achieves whole-brain coverage in approximately 6 s. However, this speed is at the expense of spatial resolution and increased susceptibility-related distortion.

**Figure 2:**
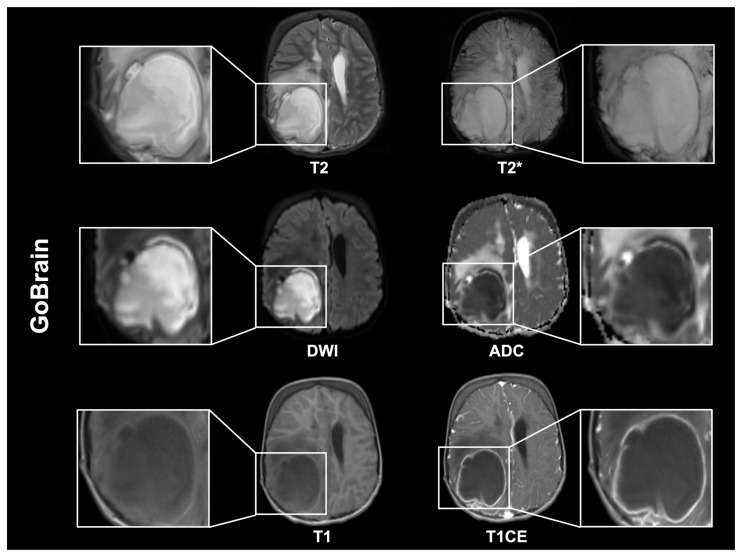
An MRI scan was performed on a 9-year-old male patient using the GOBrain protocol for the management of a brain abscess that required intensive care. Brain abscesses are focal central nervous system (CNS) infections that most commonly result from hematogenous spread, direct extension from adjacent infections (e.g., otitis or sinusitis), or occur after trauma or neurosurgical procedures. The natural progression of the condition is from the initial stage of cerebritis to the formation of a mature capsule. The MRI appearance varies according to the stage of the disease. Early lesions are characterized by poor margination, high signal intensity on T2-weighted images (T2), and the presence of diffusion restriction. In the mature stage, a well-defined capsule is evident, frequently T2w hypointense, with a centrally hyperintense core and marked diffusion restriction, manifesting as bright on DWI with low ADC due to viscous purulent contents. T2*-weighted sequences have been shown to exhibit a characteristic dual-rim sign, thus facilitating the differentiation of abscesses from glioblastomas [20,21]. On non-contrast T1-weighted images, the center is hypointense, whereas post-contrast images typically demonstrate ring enhancement.

**Figure 3:**
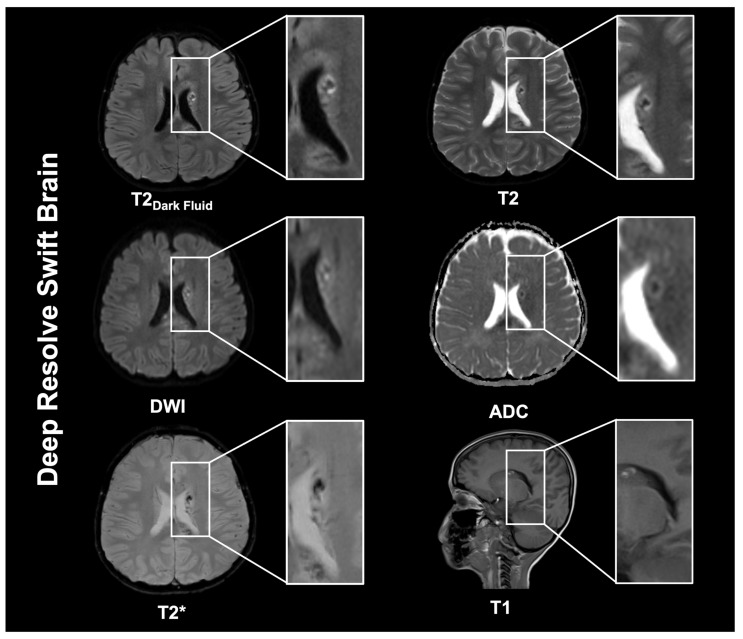
This 6-year-old male patient sustained a traumatic diffuse axonal injury (DAI). The patient was imaged using the Deep Resolve Swift Brain protocol. DAI is the result of rotational or acceleration–deceleration forces that produce widespread microscopic axonal damage, most commonly at the gray–white matter junction, within the corpus callosum, and in the brainstem [22,23]. In the early non-hemorrhagic phase, MRI can appear unremarkable; however, T2-weighted sequences typically demonstrate hyperintense foci. T2*-weighted imaging is particularly sensitive for identifying punctate microhemorrhages at these characteristic locations. DWI has also been shown to demonstrate diffusion restriction consistent with cytotoxic edema. The burden and anatomical distribution of lesions have been shown to serve as significant prognostic indicators, with brainstem involvement, particularly in deeper structures, correlating with poorer clinical outcomes. In contrast to the GOBrain protocol, the Deep Resolve Swift Brain protocol used in this case combines several multi-shot EPI (msEPI) acquisitions with DL-based reconstructions, enabling faster MRI acquisition. ssEPI has enabled ultrafast multi-contrast examinations in 1–2 min [24,25]. In ssEPI, all k-space lines are acquired following a single excitation, referred to as a “shot”. This approach enables high-speed data acquisition but requires longer readout times and longer echo times. As a result, ssEPI is prone to susceptibility-related geometric distortion, signal dropout, and pileup artifacts. To take advantage of the efficiency of EPI while mitigating signal-to-noise (SNR) and susceptibility-induced losses to image quality, Deep Resolve Swift Brain has been developed using a msEPI-based protocol that leverages a DL-based image reconstruction to provide high SNR, to reduce noise and residual aliasing during the reconstruction of T2 Dark Fluid, T2w, T2*, and T1w, complemented by ssEPI diffusion imaging within a 2-min exam [26]. In msEPI, readout lines are interleaved across multiple shots, shortening echo times and reducing geometric distortion. The sequences use an image reconstruction algorithm that integrates DL prior into an iterative SENSE reconstruction [9,27]. After an initial reconstruction, aliasing and noise are progressively reduced by repeatedly passing the image through a deep neural network, followed by a data-consistency step that enforces agreement with the measured k-space data and acquisition physics. The resulting image is then further refined using a second iterative SENSE reconstruction, yielding the DL-SENSE hybrid reconstruction [26]. By doing so, an entire axial FLAIR scan can be acquired in less than a minute. Although the EPI-based FLAIR technique is more prone to susceptibility artifacts, the methods integrated into the Deep Resolve Swift Brain protocol can partially compensate for this by performing a static field correction using a pre-scanned B_0_ map. Schuhholz et al. evaluated the EPI-based FLAIR technique for lesion detection in patients with multiple sclerosis, comparing its performance with that of conventional protocols [28]. Lesion conspicuity with EPI-based FLAIR was comparable across most brain regions, with improved conspicuity in the occipital lobe but reduced conspicuity in central regions. They further reported location-dependent limitations in SNR and contrast-to-noise ratio (CNR), as well as artifacts including spatial distortions. T2-weighted and T2*-weighted images are generated from dual-echo msEPI, yielding two images: a spin-echo-based T2-weighted image and a gradient-echo-based T2*-weighted image from a single scan. This approach thus halves the time required for image acquisition compared to running each sequence separately. The T1-weighted sequence obtained through the Deep Resolve Swift Brain protocol uses a 2D fast low-angle shot (FLASH) gradient-echo (GRE) sequence optimized for speed while maintaining essential anatomical detail. This rapid acquisition inherently comes with compromises, such as a lower baseline SNR and artifacts, e.g., Gibbs ringing or undersampling. However, the integrated Deep Resolve DLR enhances image quality by suppressing noise amplification typically caused by aggressive acceleration, and by restoring sharpness and detail. Prior research has shown that DLR images exhibit lower noise levels than those obtained using traditional full-scan techniques. This trait can make DLR images appear unnatural to experienced radiologists. The DLR algorithm might produce images in which the internal tissue structure (e.g., of a tumor) appears more uniform and exhibits softer contrast than conventional images. Additionally, individual preferences and familiarity with the types of images commonly seen in regular practice also influence these differing evaluations [29]. In contrast, DLR can result in inferior anatomical delineation, as demonstrated in a recent neuroradiological study of the hippocampus, brainstem, and cerebellum [30]. DL-based reconstructions might also exhibit artifacts mistaken for lesions due to instabilities [30,31]. Therefore, large comparative studies are still needed to establish diagnostic equivalence with standard protocols, especially for subtle alterations such as cortical malformations and other small or low-contrast enhancing abnormalities, and to determine the precise clinical indications, staff training, reliability, and reproducibility. There are contexts in which specific, high-resolution MRI protocols are still required, such as in neuronavigation and epilepsy. Accelerated pediatric protocols such as GOBrain and Deep Resolve Swift Brain may not be adequately suited for these purposes.

**Figure 4:**
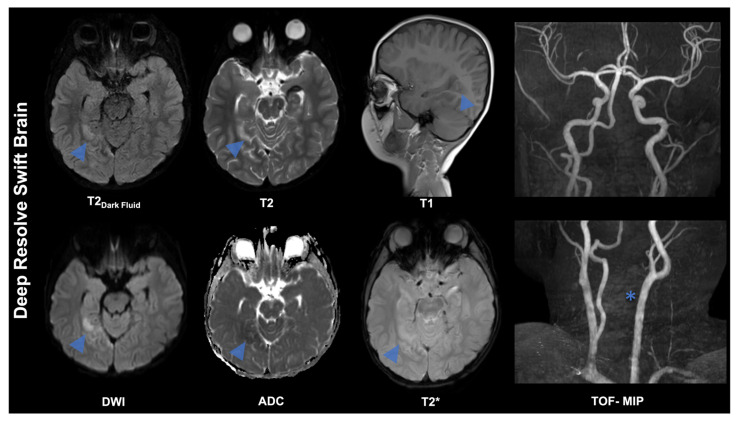
A 4-year-old boy was diagnosed with a left vertebral artery dissection after neck trauma, leading to a posterior circulation stroke. Vertebral artery dissection most commonly presents with neck pain and headache, often accompanied by brainstem or cerebellar symptoms. Using the Deep Resolve Swift Brain MRI protocol, DWI demonstrates infarcts in the right posterior circulation (blue arrow). The DWI component of the Deep Resolve Swift Brain protocol is optimized for rapid brain imaging while maintaining high sensitivity for diffusion restriction. Although based on conventional ssEPI, it incorporates contemporary acceleration methods that markedly shorten acquisition time without materially compromising signal-to-noise ratio. It is important to note that the protocol utilizes simultaneous multi-slice (SMS) imaging. This technique enables simultaneous excitation and acquisition of multiple slices, unlike the conventional sequential approach. To illustrate, an SMS factor of 2 signifies that two anatomical slices are acquired in parallel during each TR. During reconstruction, slice-specific coil sensitivity profiles are employed; these are known as slice-GRAPPA. The purpose is to separate signals that are acquired simultaneously and overlap, since each slice is encoded with a distinct modulation pattern. This approach has been shown to approximately double acquisition speed without lengthening the echo train or TR. Furthermore, in-plane parallel imaging (typically GRAPPA with an acceleration factor of 2) is employed to reduce the number of phase-encoding steps, thereby shortening the echo train and decreasing T2-related blurring and susceptibility artefacts. The use of this dual-acceleration strategy enables the acquisition of diffusion-weighted images at b ≈ 1000 s/mm^2^ (in conjunction with a reference image at b = 0) within a single shot per SMS group, thereby achieving a substantial reduction in acquisition time compared with conventional ssEPI DWI at comparable resolution. Notwithstanding the reduced scan duration, the images remain adequate for diagnostic purposes. Research has demonstrated that SMS-accelerated DWI can be employed to achieve diagnostic performance comparable to that of conventional acquisitions whilst simultaneously reducing scan time [32]. A recognized limitation of ssEPI DWI is geometric distortion, which becomes more pronounced in regions exhibiting magnetic field inhomogeneity. The Deep Resolve Swift Brain protocol has been developed to address this issue by incorporating a static B0 field map into the reconstruction process. This allows for the identification and correction of susceptibility-related stretching and compression, thereby enhancing anatomical fidelity. Furthermore, the DWI data can be reconstructed using Deep Resolve deep learning to enhance the SNR while maintaining spatial resolution, without being constrained by conventional acceleration trade-offs. Finally, maximum-intensity projection (MIP) images from an additional time-of-flight (TOF) MR angiography (MRA) acquisition—performed outside the Deep Resolve Swift Brain protocol—demonstrate signal loss and occlusion of the left vertebral artery (*), consistent with dissection.

**Figure 5:**
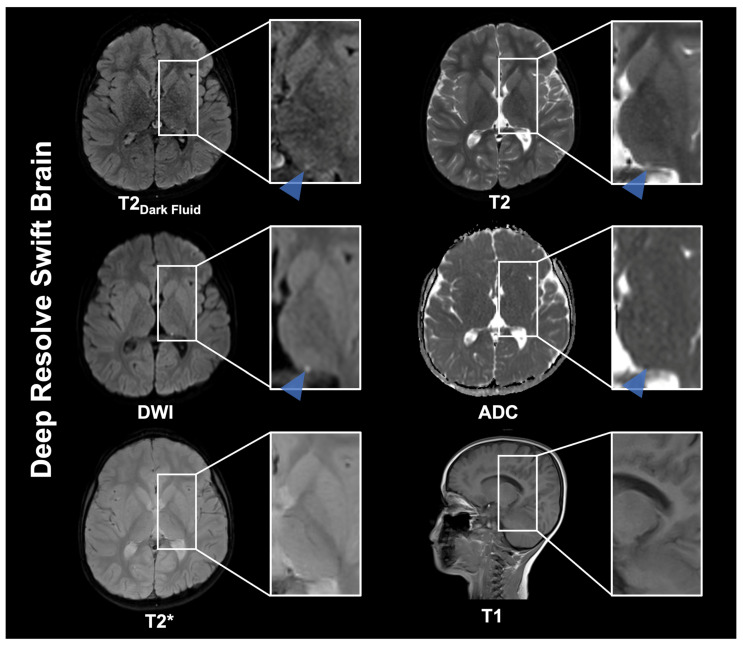
Presents additional images from the Deep Resolve Swift Brain protocol in the same 4-year-old boy, who developed a left vertebral artery dissection after neck trauma, resulting in a posterior circulation stroke. The protocol includes axial T2-weighted dark-fluid, T2-weighted, diffusion-weighted, and T2*-weighted sequences, as well as a sagittal T1-weighted sequence. A T2 well-defined punctate ischemic lesion in the left posterior thalamus is evident on diffusion-weighted images with a corresponding hypointense signal on the ADC map.

**Figure 6:**
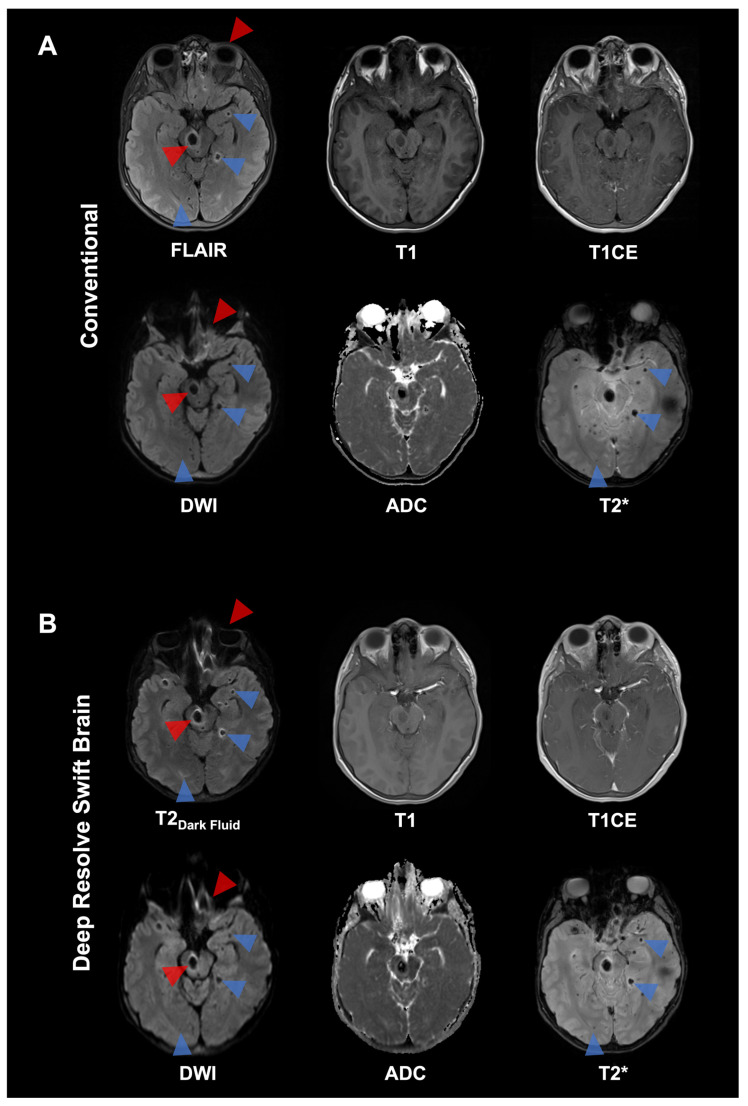
This 13-year-old male patient with a history of Ewing’s sarcoma presents with a leukostasis syndrome as a rare emergency related to hyperleukocytosis leading to microvascular occlusion and central nervous system (CNS) ischemia and bleeding (blue arrowheads). The top rows (**A**) show conventional MRI sequences, whereas the bottom rows (**B**) show the corresponding accelerated sequences using Deep Resolve Swift Brain. Due to alleviated EPI-related susceptibility artifacts and image distortions in T2-weighted dark fluid and DWI (red arrowheads), image quality is partially reduced in the Deep Resolve Swift Brain protocol in direct comparison. Brain MRI reveals small diffusion-restricted infarcts or microhemorrhages on T2*. CNS involvement can cause subtle signal changes that require careful review of DWI and T2* sequences (blue arrowheads) to detect early microinfarcts and -bleeds, which might be less visible on standard FLAIR and accelerated T2-weighted dark fluid sequences of the Deep Resolve Swift Brain protocol.

**Figure 7:**
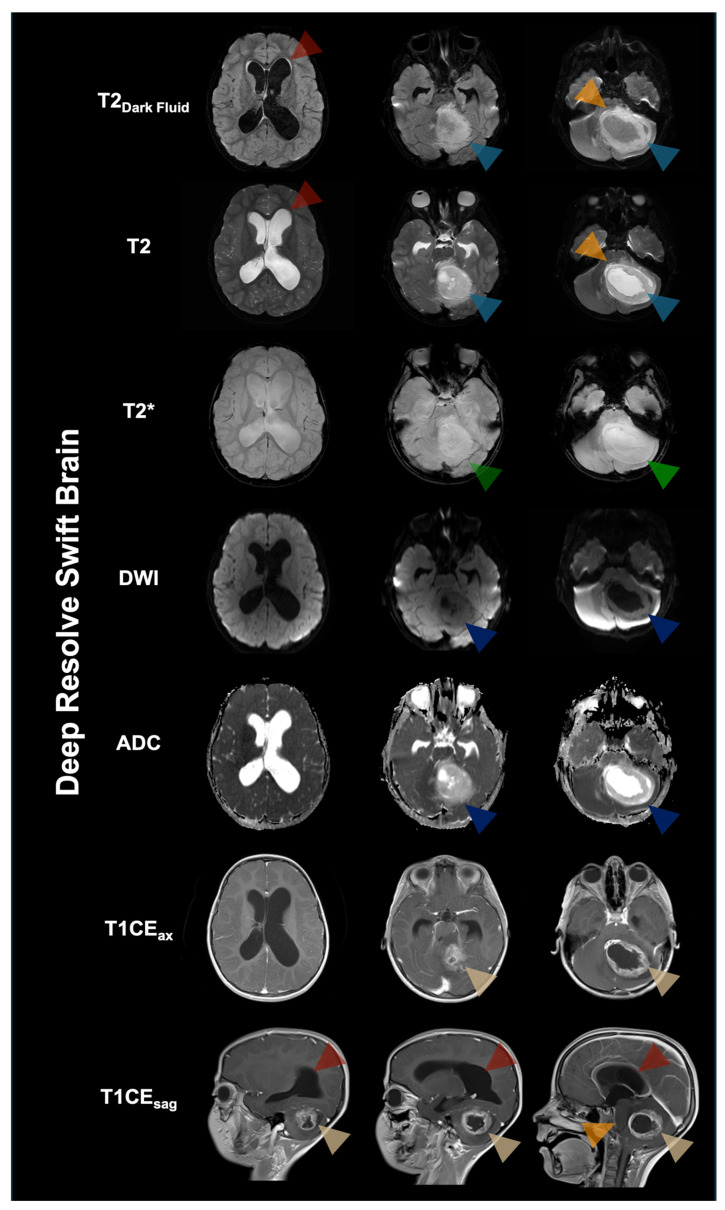
A 6-year-old female patient was admitted with subacute clinical deterioration, presenting with fasting vomiting and headaches. Brain MRI performed with the Deep Resolve Swift Brain protocol demonstrated a large posterior fossa tumor occupying a large portion of the left cerebellar hemisphere and the vermis, with secondary congestion. Given the marked mass effect and obstructive hydrocephalus, urgent surgery was performed for tumor decompression and placement of a ventricular drain. Infratentorial tumors account for 45–60% of brain tumors in children, including medulloblastoma (40%), pilocytic astrocytoma (30%), ependymoma, embryonal tumors with multilayered rosettes, diffuse midline glioma, and diffuse leptomeningeal glioneural tumor [33]. The tumor in this case is a histopathology-confirmed WHO grade 1 pilocytic astrocytoma. Despite the growing integration of molecular and genetic profiling into the diagnostic workup of these tumors, they continue to exhibit characteristic imaging features that can facilitate differentiation among tumor entities [34,35]. The presented pilocytic astrocytoma shows typical changes on different contrast levels. On T2-weighted images, the mass is hyperintense to the adjacent brain parenchyma and surrounded by edema (light blue arrowhead). Anterior displacement of the brainstem and compression of the fourth ventricle (orange arrowheads) resulted in secondary enlargement of the supratentorial ventricular system with signs of transependymal CSF flow (red arrowheads). The lesion showed mixed solid and cystic components, with contrast enhancement indicating blood–brain barrier disruption on axial (ax) and sagittal (sag) post-contrast T1-weighted images (T1CE; yellow arrowheads). DWI also delineated the solid and non-solid tumor portions (dark blue arrowheads). No intralesional hemorrhage or calcification was detected on T2* imaging (green arrowheads). Although the protocol includes partially EPI-based sequences, artifacts remained limited, and diagnostic image quality was preserved despite the infratentorial location. In this pediatric emergency-focused case series, GOBrain and Deep Resolve Swift Brain enabled comprehensive multi-contrast brain MRI within markedly reduced acquisition times while maintaining diagnostically useful image quality across a range of clinically relevant pathologies. These protocols may serve as practical adjuncts for rapid triage and time-critical decision-making, potentially reducing motion-related nondiagnostic scans and the need for sedation in selected scenarios, while not fully replacing conventional, indication-tailored high-resolution examinations. Future larger studies with standardized reference protocols, blinded reader assessments, and quantitative performance measures are warranted to define optimal indications, limitations, and implementation requirements in routine pediatric neuroimaging.

## Data Availability

The original contributions presented in this study are included in the article/Supplementary Material. Further inquiries can be directed to the corresponding author.

## Supplementary Materials

The following supporting information can be downloaded at: https://www.mdpi.com/article/10.3390/diagnostics16050681/s1, Table S1: MRI acquisition parameters of GOBrain and Deep Resolve Swift Brain protocols at 3 Tesla. Table S2. Case overview using GOBrain and Deep Resolve Swift Brain protocols at 3 Tesla.

## Abbreviations

The following abbreviations are used in this manuscript:

ADC	Apparent Diffusion coefficient
CE	Contrast-enhanced
CNS	Central nervous system
CR	Conventional reconstruction
DL	Deep-learning
DLR	Deep-learning reconstruction
DWI	Diffusion-weighted imaging
EPI	Echo-planar imaging
GRAPPA	GeneRalized Autocalibrating Partial Parallel Acquisition
MIP	maximum intensity projection
MRI	Magnetic resonance imaging
ms	Multi-shot
PI	Parallel imaging
SNR	Signal-to-noise ratio
SMS	simultaneous multi-slice
ss	single-shot
TOF	Time-of-flight
TR	Time of repetition
TSE	Turbo spin-echo
