# Supplementary material for: Insights into Accelerated MRI Protocols for Pediatric Brain Assessment in Emergency Cases
Source: Diagnostics (Basel). 2026 Feb 26;16(5):681. doi: 10.3390/diagnostics16050681 (PMC12984488; doi:10.3390/diagnostics16050681)
Supplement: Supplementary file 1 [file diagnostics-16-00681-s001.zip › diagnostics-4116585-supplementary.pdf]

Table S1. MRI acquisition parameters of GOBrain and Deep Resolve Swift Brain protocols at 3 Tesla.

Parameters	GOBrain					Deep Resolve Swift Brain				
	**T2**$_{TSE\ Dark\ Fluid}$	**T2**$_{TSE}$	**T2***$_{ep2d}$	**T1**$_{GRE\ FLASH}$	**DWI**$_{ep2d+}$	**T2**$_{msEPI\ Dark\ Fluid}$	**T2**$_{msEPI}$	**T2***$_{msEPI}$	**T1**$_{msEPI}$	**DWI**$_{SMS\text{-}ssEPI+}$
**Field of view (mm)**	220	220	220	220	240	230	230	230	230	230
**Voxel size (mm)**	0.9 x 0.9 x 5.0	0.9 x 0.9 x 5.0	0.9 x 0.9 x 5.0	0.9 x 0.9 x 4.0	1.5 x 1.5 x 5.0	0.9 x 0.9 x 4.0	0.9 x 0.9 x 4.0	0.9 x 0.9 x 4.0	0.9 x 0.9 x 4.0	0.7 x 0.7 x 4.0
**Slice thickness (mm)**	5	5	5	4	5	4	4	4	4	4
**Number of slices**	25	25	25	35	31	32	32	32	33	32
**Distance Factor (%)**	20	20	20	20	12	20	20	20	20	20
**Base Resolution**	256	256	256	256	160	256	256	256	256	160
**Parallel imaging factor**	2	3	2	2	2	2	2	2	3	2x2
**Acceleration mode**	GRAPPA	GRAPPA	GRAPPA	GRAPPA	GRAPPA	GRAPPA	GRAPPA	GRAPPA	GRAPPA	GRAPPA
**TR (ms)**	8000	6200	589	240	4200	8000	4390	4390	211	3000
**TE (ms)**	119	78	19.80	2.46	72	93	20.30	93	2.46	68
**Averages**	1	1	1	1	1	1	1	1	1	1
**Concatenations**	2	2	1	1	1	2	1	1	1	1
**Acquisition time (min)**	1:52	1:02	1:13	0:41	1:07	0:51	0:25	0:00#	0:21	0:21
**Time savings using Swift Brain vs. GO Brain sequences in min (%)**						1:01 (54 %)	0:37 (60 %)	1:13 (100 %)	0:20 (49 %)	0:46 (79 %)
**Time-saving on average in min (%)**			ʻ					0:47 (86 %)		
**Total time saving in min**								3:57		

w = weighted; GRAPPA = GeneRalized Autocalibrating Partial Parallel Acquisition (parallel imaging technique); TR = repetition time; TE = echo time; TSE = turbo spin echo; mm = millimeter; ms = millisecond; min = minute; +b-values are 0 and 800 s/mm2 for Go Brain protocol and 0 and 1000 s/mm2 for Swift Brain protocol; #T2* data is aquired in the T2w msEPI sequence.

Table S2. Case overview using GOBrain and Deep Resolve Swift Brain protocols at 3 Tesla.

Case	Description	MRI protocol
1	Acute lymphoblastic leukemia (ALL)	GoBrain
2	Brain abcess	GoBrain
3	Stroke (vertebral artery dissection)	Deep Resolve Swift Brain protocol
4	Traumatic diffuse axonal injury (DAI).	Deep Resolve Swift Brain protocol
5	Leukostasis syndrome	Deep Resolve Swift Brain protocol
6	Posterior fossa tumor (WHO grade 1 pilocyctic astrocytoma)	Deep Resolve Swift Brain protocol
